# Human Papillomavirus 16 Lineage D is Associated with High Risk of Cervical Cancer in the Brazilian Northeast Region

**DOI:** 10.1055/s-0043-1772180

**Published:** 2023-09-08

**Authors:** Luís Felipe Leite Martins, Miguel Ângelo Martins Moreira, Rodrigo Alves Pinto, Neilane Bertoni dos Reis, Shayany Pinto Felix, João Paulo Castello Branco Vidal, Leuridan Cavalcante Torres, Ariani Impieri Souza, Liz Maria de Almeida

**Affiliations:** 1 Cancer Surveillance and Data Analysis Division, Instituto Nacional de Câncer, Rio de Janeiro, RJ, Brazil; 2 Tumor Genetics and Virology Program, Instituto Nacional de Câncer, Rio de Janeiro, RJ, Brazil; 3 Instituto de Medicina Integral Prof. Fernando Figueira, Recife, PE, Brazil; 4 Division of Population Research, Instituto Nacional de Câncer, Rio de Janeiro, RJ, Brazil; 5 Universidade Federal do Maranhão, São Luis, MA, Brazil; 6 Coordination of Prevention and Surveillance, Instituto Nacional do Câncer, Rio de Janeiro, RJ, Brazil

**Keywords:** HPV16, lineage A, lineage D, CIN2/CIN3, cervical cancer, HPV16, linhagem A, linhagem D, NIC2/NIC3, câncer colo útero

## Abstract

**Objective** Similar to Human Papillomavirus (HPV) genotypes, different lineages of a genotype also have different carcinogenic capabilities. Studies have shown that specific genotype lineages of oncogenic HPV are associated with variable risks for the development of cervical intraepithelial neoplasia (CIN2/CIN3) and cervical cancer. The present study aimed to analyze the genetic diversity of the HPV16 genotype in women with CIN2/CIN3 and cervical cancer, from the northeast region of Brazil.

**Methods** A cross-sectional multicenter study was conducted in the northeast region of Brazil, from 2014 to 2016. This study included 196 cases of HPV16 variants (59 and 137 cases of CIN2/CIN3 and cervical cancer, respectively). The difference of proportion test was used to compare patients with CIN2/CIN3 and cervical cancer, based on the prevalent HPV16 lineage ( *p* < 0.05).

**Results** According to the histopathological diagnosis, the percentage of lineage frequencies revealed a marginal difference in the prevalence of lineage A in CIN2/CIN3, compared with that in cervical cancer ( *p* = 0.053). For lineage D, the proportion was higher in cancer cases (32.8%), than in CIN2/CIN3 cases (16.9%), with *p* = 0.023.

**Conclusion** HPV16 lineage A was the most frequent lineage in both CIN2/CIN3 and cervical cancer samples, while lineage D was predominant in cervical cancer, suggesting a possible association between HPV16 lineage D and cervical cancer.

## Resumo

**Objetivo** Tanto os tipos quanto as linhagens do Papilomavírus Humano (HPV) parecem ter diferentes capacidades carcinogênicas e estão associados a riscos variados para o desenvolvimento de neoplasia intraepitelial cervical (NIC) e câncer de colo do útero. O presente estudo tem como objetivo analisar a diversidade genética do genótipo HPV 16 nos casos de NIC2/NIC3 e câncer de colo de útero em mulheres da região Nordeste do Brasil.

**Métodos** Estudo transversal de base hospitalar realizado na região Nordeste do Brasil no período de 2014 a 2016. A amostra foi composta por 196 casos da variante HPV-16 (59 casos de NIC2/NIC3 e 137 de câncer do colo do útero). O teste de diferença de proporção foi usado para comparar os grupos NIC2/NIC3 e câncer de colo do útero por linhagem viral em relação à prevalência da linhagem HPV-16. Foi considerada significância estatística o valor de *p* < 0,05.

**Resultados** As frequências de linhagem por diagnóstico histopatológico mostraram diferença limítrofe da linhagem A no grupo NIC2/NIC3 em relação ao grupo câncer de colo de útero ( *p* = 0,053). Por outro lado, em relação à linhagem D, houve uma proporção maior nos casos de câncer (32,8%) quando comparado ao grupo NIC2/NIC3 (16,9%) e esta diferença se mostrou estatisticamente significante ( *p* = 0,023).

**Conclusão** A linhagem A do HPV-16 foi a mais frequente tanto nas amostras CIN2/CIN3 quanto nas amostras de câncer de colo de útero, enquanto a linhagem D predominou no câncer de colo do útero, sugerindo uma possível associação da linhagem D de HPV-16 com câncer de colo de útero.

## Introduction

Cervical cancer is the fourth most common cancer, in terms of incidence and mortality, among women worldwide. 1 In Brazil, it is the third and fourth most common cancer among women, in terms of incidence and mortality, respectively. 2 3 Several studies have shown that the Human Papillomavirus (HPV) is a predominant, but not the only, factor for cervical cancer development. The viral genotype HPV16 is the most prevalent in cervical cancer and is considered a Group 1 carcinogenic agent for humans by the International Agency for Cancer Research. 4 The study of the genetic diversity of HPV16 has enabled the characterization of specific viral lineages associated with a higher risk of cervical intraepithelial neoplasia (CIN2/CIN3) and cervical cancer development. 5 6 7 8 For HPV16, the lineages were initially named according to their geographical prevalence and separated into five groups: European (EUR), Asian (As), Asian-American (AA), African 1 (Af1), and African 2 (Af2). 9 10 11 12 In 2013, Burk et al., proposed a new α-numeric nomenclature for all HPV lineages based on the differences in the complete viral genome sequence. 13 The lineages for HPV16 were renamed as follows: A (corresponding to EUR and As), B (corresponding to Af1), C (corresponding to Af2), and D (corresponding to AA).

Studies have shown that HPV16 lineages B, C, and D with higher pathogenicity are associated with a higher viral persistence, compared with the lineage A. 4 14 15 Additionally, the HPV16 lineages B, C, and D are also associated with a higher risk of CIN2/CIN3 and cervical cancer. 14 16 17 The D lineage is also associated with adenocarcinoma. 18 A longitudinal study has shown that the HPV16 sub-lineages D2 and D3 are more significantly associated with CIN2/CIN3 and cervical cancer, compared with other lineages/sub-lineages. 16 19 Similar characteristics were reported in lineages of other HPV genotypes, such as HPV 18 and HPV 45. 20

In this study, we analyzed the genetic diversity of the HPV16 genotype in CIN2/CIN3 and cervical cancer cases in women from the northeast region of Brazil. In this region, cervical cancer was ranked second in terms of cancer incidence, excluding nonmelanoma skin cancer (20.48/100,000), 2 and first in terms of mortality due to cancer (9.52/100.000) among women, in 2020. 3 The present work was part of a multicenter study on the HPV genotypes prior to the introduction of the HPV vaccine in the National Immunization Program, which was performed in two other cities in Brazil (Rio de Janeiro and Belém). 21 22

## Methods

A cross-sectional multicenter study was performed at two hospitals for cancer treatment in Cidade do Recife, Pernambuco, Brazil, between July 2014 and December 2016.

Women ≥18 years old, with pap smear tests indicative of high-grade intraepithelial lesion (HGL; CIN2/CIN3) or cervical cancer, and who were referred for a colposcopy exam, were invited to participate in the present study. Women who underwent a biopsy and were diagnosed for CIN2/CIN3 or cervical cancer, based on histopathological examination, were included in this analysis. The exclusion criteria included women previously treated for cervical cancer (cancer surgery, radiotherapy, or chemotherapy) or those with cognitive or physical disabilities that could prevent them from answering a questionnaire.

After receiving signed consent, the women were interviewed by trained nurses using an epidemiological questionnaire. We collected data about their socioeconomic status, knowledge about cervical cancer prevention, access to healthcare services for diagnosis and treatment, hormonal and reproductive histories, and tobacco usage. Additional clinical information was obtained from their medical records. The biopsy samples were stored in RNALater until nucleic acid isolation, and were then sent to the research laboratory of the hospital.

DNA was isolated from the biopsy samples using the QIAamp DNA Mini Kit (Qiagen; Cat. Number 51306, North Rhine-Westphalia, Germany). HPV was detected through PCR amplification using the primer sets PGMY07 and PGMY09, 23 whereas reactions without PCR products underwent nested PCR, using the primers GP5 +/GP6 + . 24 Samples negative for HPV DNA amplification after nested PCR were subjected to PCR for β-globin, and samples positive for β-globin and negative for HPV through nested PCR were considered negative for HPV. For HPV identification, the PCR products were purified using the Illustra GFX PCR DNA and Gel Band Purification Kit (GE Healthcare; Cat. Number 28403471, Buckinghamshire, UK), and were further subjected to DNA sequencing in both directions, using the Big Dye Terminator Cycle Sequencing Ready Reaction V3.1 Kit (Applied Biosystems; Cat. Number 4336919, Texas, USA), as per manufacturer's instructions, and sequenced in an ABI 3730xL DNA Analyzer (Applied Biosystems, Osaka, Japan). The electropherograms of each sample were checked manually and a consensus sequence of the bidirectional sequencing was subjected to HPV genotype identification using the Blast software ( https://blast.ncbi.nlm.nih.gov/Blast.cgi). ^25^

Among the 415 samples evaluated for HPV genotyping through DNA sequencing, the 8 most common types were: HPV16 (58.6%), HPV45 (7.2%), HPV18 (7.0%), HPV35 (4.6%), HPV58 (3.6%), HPV31 (3.1%), HPV33 (2.7%), and HPV52 (2.4%). HPV16 was the most common HPV genotype in CIN2/CIN3 (53.9%) and cervical cancer (60.7%). A total of 243 samples contained the HPV16 genotype, and 196 samples (80.7%) were further assessed to identify their lineages.

Samples exhibiting the HPV16 genotypes were subjected to PCR amplification of the viral genomic regions *LCR* and *E6,* in two overlapping fragments, as previously described. 26 The resultant sequence, obtained by sequencing the overlapping fragments, was aligned to HPV16 lineage reference sequences suggested by Burk et al. 13 HPV16 lineage identification was performed by detecting a sequence signature (the presence of specific nucleotides at specific sequence positions), as previously described. 12 HPV16 lineage nomenclature used in this study followed those provided by Burk et al. 13

The epidemiological data were stored using Epi Info 3.5.1 and then linked to both the clinical and HPV DNA sequence data. The final database was analyzed using Stata v.15.0. the chi-squared test (or the Fisher exact test) was used to compare the distribution of the patients based on selected characteristics, according to their histopathological diagnosis. The ratio difference test was used to compare the prevalence of HPV16 viral strains, considering two groups: CIN2/CIN3 and cervical cancer ( *p* < 0.05). The B and C lineages were grouped due to low frequency.

All study procedures were approved by the Institutional Ethics Committees at both hospitals (CAAE 24687713.8.0000.5201 and CAAE 40349014.0.0000.5205).

## Results

Selected characteristics of the 196 female patients included in the present study, according to disease status (CIN2/CIN3 versus cervical cancer), are presented in Table 1 . We observed that the women diagnosed with CIN2/CIN3 were younger than those diagnosed with cervical cancer. No statistical differences were found between the women with CIN2/CIN3 and cervical cancer for oral contraceptive use or tobacco exposure. The number of childbirths was greater in women with cervical cancer than in those with CIN2/CIN3.

**Table 1:** Distribution of female patients based on selected characteristics, according to their histopathological diagnosis

Variable	CIN2/CIN3		Cervical cancer		***p-value*** *
	*n* = 59	%	*n* = 137	%	
Age (years old)					
20–39	37	62.7	43	31.4	< 0.001
40–49	16	27.1	40	29.2	
50–59	6	10.2	23	16.8	
≥ 60	0	0.0	31	22.6	
Number of childbirths					
None	3	5.1	3	2.2	
1–2	28	47.5	43	31.4	0.07
3–4	17	28.8	44	32.1	
5–6	5	8.5	14	10.2	
≥ 7	6	10.1	33	24.1	
Oral contraceptive use					
Yes	42	71.2	88	64.2	0.34
No	17	28.8	49	35.8	
Tobacco exposure					
Yes	26	44.1	76	55.5	0.14
No	33	55.9	61	44.5	

Abbraviation: CIN, cervical intraepithelial neoplasia.

(*) Chi-squared test or Fisher exact test.

The LCR and E6 regions of each sample with an HPV16 genotype were aligned with HPV16 reference sequences representing the lineages A, B, C, and D, and the presence of specific SNPs was used to identify HPV16 lineages present in each sample. The distribution of the HPV16 lineages is as follows: lineage A, 130 women; lineage B, 1 woman; lineage C, 10 women; and lineage D, 55 women. We observed distinct frequencies of HPV16 lineages between CIN2/CIN3 and cervical cancer, with lineage A being more frenquent in CIN2/CIN3 ( *p* = 0.053). Moreover, based on the histopathological diagnosis, the comparison of HPV16 lineage frequencies showed a higher proportion of the D lineage in cervical cancer than that in CIN2/CIN3 ( *p* = 0.023) ( Table 2 ).

**Table 2:** Distribution of female patients, according to HPV16 lineage frequencies and their histopathological diagnosis

HPV16 lineages	Histopatological diagnosis					***p-value*** *
	Total	CIN2/CIN3		Cervical Cancer		
	*n* (%)	*n*	%	*n*	%	
**A**	130 (66.3)	45	76.3	85	62.0	0.053
**B/C**	11 (5.6)	4	6.8	7	5.1	0.635
**D**	55 (28.1)	10	16.9	45	32.8	0.023

*Test for proportion difference among patients with CIN2/CIN3 and cervical cancer.

## Discussion

The lineage A of HPV16 was the most frequent in the samples examined in the present study, for both CIN2/CIN3 and cervical cancer, while lineage D was predominant in cervical cancer samples, suggesting an association between the lineage D of HPV16 and cervical cancer.

The genetic variations in HPV16 can influence the risk of cervical cancer, which vary based on the different lineages or sublineages present in different regions of the world. 8 Previous studies attempted to demonstrate an association between non-European lineages (B/C/D) and a higher risk for developing CIN2/CIN3 14 17 and cervical cancer, 17 27 compared with the European lineage (A). A major limitation of these studies was the grouping of lineages B, C, and D as non-European lineages, which did not allow the differentiation of the carcinogenic potential among these lineages. A recent study on > 3,200 women, comparing women without lesions and with CIN1 and women with CIN2 or CIN3 or invasive cancer, have reported an association between the HPV16 sub-lineages D2 and D3 and CIN3 and/or cervical cancer. 17 Clifford et al. analyzed samples from > 7,100 women, comparing those with normal cells, or atypical squamous cells of undetermined significance, or low-grade squamous intraepithelial lesions or CIN1, with those with invasive cervical cancer, and found an association between the HPV16 lineage D and sublineage A4 and cancer diagnosis. 8

A recent study on Croatian women confirmed that HPV16, mainly belonging to the European branch, was most frequently associated with histologically confirmed high-grade intraepithelial lesions (CIN2 or CIN3) and cervical cancer. 28

In the present study, the lineage A of HPV16 was the most frequently detected lineage, and although it was associated with high-grade lesions (CIN2/CIN3), we could not demonstrate a difference between these high-grade lesions and cervical cancer. Another study conducted in a different region in Brazil also found a high prevalence of the lineage A of HPV16 in samples of high- and low-grade lesions, including cervical cancer. 29

In contrast, the D lineage of HPV16 showed a significant association with cervical cancer, compared with high-grade lesions, suggesting that the lineage D of HPV16 is involved in the progression of HPV16 infection to cervical cancer. Although this is a cross-sectional study, the present study provides novel data on the association between specific lineages of HPV16 and cervical cancer. Our data suggest that the lineage D could be more aggressive with respect to the progression of high-grade lesions into cancer, without restricting the association from linking normal/low grade lesions and invasive cancer.

## Conclusion

The A lineage of HPV16 was the most frequent in both the CIN2/CIN3 and cervical cancer samples, whereas the lineage D of HPV16 was predominant in cervical cancer, suggesting an association between HPV16 lineage D and cervical cancer.
